# Orientation of DNA Minicircles Balances Density and Topological Complexity in Kinetoplast DNA

**DOI:** 10.1371/journal.pone.0130998

**Published:** 2015-06-25

**Authors:** Yuanan Diao, Victor Rodriguez, Michele Klingbeil, Javier Arsuaga

**Affiliations:** 1 Department of Mathematics and Statistics, University of North Carolina at Charlotte, Charlotte, North Carolina, United States of America; 2 College of Physicians and Surgeons, Columbia University, New York, New York, United States of America; 3 Department of Microbiology, University of Massachusetts, Amherst, Massachusetts, United States of America; 4 Department of Mathematics, University of California at Davis, Davis, California, United States of America; 5 Department of Molecular and Cellular Biology, University of California at Davis, Davis, California, United States of America; Universidad de Chile, CHILE

## Abstract

Kinetoplast DNA (kDNA), a unique mitochondrial structure common to trypanosomatid parasites, contains thousands of DNA minicircles that are densely packed and can be topologically linked into a chain mail-like network. Experimental data indicate that every minicircle in the network is, on average, singly linked to three other minicircles (i.e., has mean valence 3) before replication and to six minicircles in the late stages of replication. The biophysical factors that determine the topology of the network and its changes during the cell cycle remain unknown. Using a mathematical modeling approach, we previously showed that volume confinement alone can drive the formation of the network and that it induces a linear relationship between mean valence and minicircle density. Our modeling also predicted a minicircle valence two orders of magnitude greater than that observed in kDNA. To determine the factors that contribute to this discrepancy we systematically analyzed the relationship between the topological properties of the network (i.e., minicircle density and mean valence) and its biophysical properties such as DNA bending, electrostatic repulsion, and minicircle relative position and orientation. Significantly, our results showed that most of the discrepancy between the theoretical and experimental observations can be accounted for by the orientation of the minicircles with volume exclusion due to electrostatic interactions and DNA bending playing smaller roles. Our results are in agreement with the three dimensional kDNA organization model, initially proposed by Delain and Riou, in which minicircles are oriented almost perpendicular to the horizontal plane of the kDNA disk. We suggest that while minicircle confinement drives the formation of kDNA networks, it is minicircle orientation that regulates the topological complexity of the network.

## Introduction

Kinetoplastids are single cell flagellated protists that can be free living or parasitic. Trypanosomatids are parasitic Kinetoplastids and include those responsible for the human diseases African and American trypanosomiasis and Leishmaniasis [1, 2]. The mitochondrial DNA of these organisms, called kinetoplast DNA (kDNA), is organized into maxicircles and minicircles. Maxicircles do not encode tRNAs however they do contain ribosomal genes and other mitochondrial genes; their size ranges from 20 to 40 kb. Minicircles on the other hand range in size from 1.0 to 10 kb and code for guide RNAs that edit maxicircles transcripts [3–6].

Most Trypanosomatid parasites have catenated kDNA networks [7, 8] and it has been proposed that the transition from a non-catenated structure in free-living relatives to the catenated network may have been an important preadaptation for the success of the parasites [9], furthermore it has been argued that the disruption of kDNA structure can be used as a potential chemotherapy target ([10] and reviewed in [11]), [12]. However, the formation and maintenance of this network is still a major puzzle in spite of significant advances through numerous biochemical and molecular studies (for a review see [13]).

**Fig 1:**
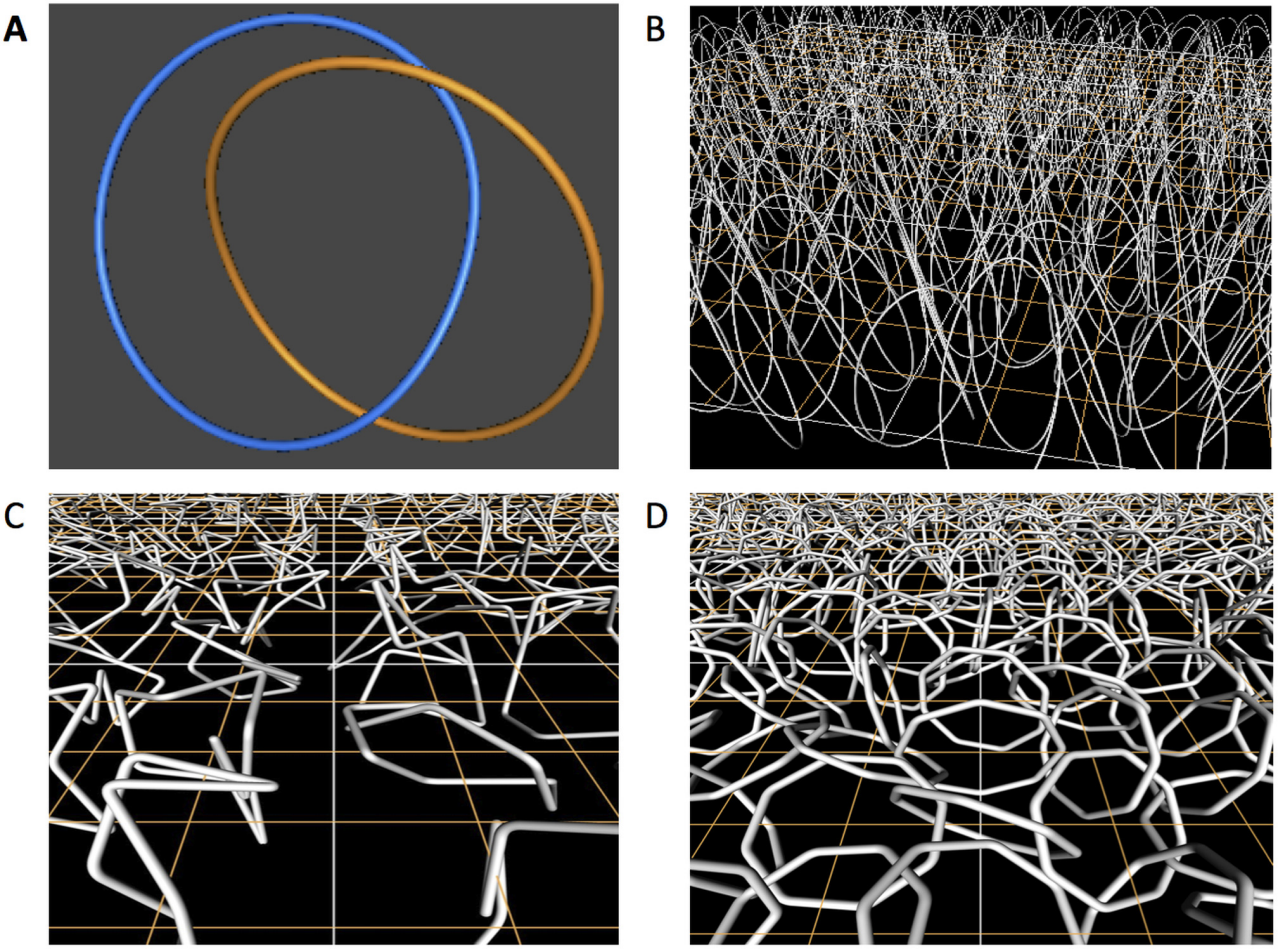
Hopf Links and Models proposed to analyze the topological properties of the minicircle network. A: A Hopf link; B: A grid of minicircles whose orientation has been biased. Volume effects are not considered and minicircle thickness is shown to help trace the trajectory of each minicircle; C: A grid of minicircles represented by freely jointed closed chains to study the effects of DNA bending; D: A grid of minicircles, represented by octagonal polygons, to study the effects of volume exclusion due to electrostatic interactions.

In the Trypanosomatid human parasites, kDNA is confined within a small cylindrical structure, called the kinetoplast disk, in which DNA concentration is comparable to that of the bacterial nucleoid [14]. Minicircles are relaxed (instead of supercoiled) and topologically linked into a planar network [15]. During the early phase of the kDNA replication cycle in *Crithidia fasciculata*, an insect parasite whose kDNA network structure has been studied in the most detail and closely resembles that of kDNA networks from human parasites, minicircles are singly linked via a Hopf link (Fig 1A) to three other minicircles (i.e. they have mean valence equal to three) [15]. The mean minicircle valence increases from three to six in kDNA S-phase returning to three prior to segregation of daughter networks [16, 17].

In our earlier work we hypothesized that volume confinement is the main contributing factor to the formation of the kDNA network. To test this hypothesis, we introduced a mathematical model in which kDNA minicircles were modeled as randomly oriented geometric circles whose centers were placed on a planar square lattice grid (Fig 1B). Our modeling showed, in agreement with experimental observations, that a network containing nearly all minicircles is the most likely conformation at high minicircle density and that the mean minicircle valence increases linearly with minicircle density [18, 19].

In this work, we first show how the predicted mean valence at the observed *in vivo* minicircle density is two orders of magnitude greater than that experimentally observed in kDNA. This surprisingly large discrepancy between the predicted and observed mean valences suggests that biophysical factors other than confinement contribute to the topological properties of the network. Type II topoisomerases (Topo II) actively simplify the topology of DNA [20] even under crowded cellular conditions and could maintain a lower mean valence. However, this biological factor seems unlikely since genetic depletion of the mitochondrial Topo II in the human parasite *Trypanosoma brucei* results in networks with simplified topologies [21, 22]. We therefore hypothesize that other biophysical factors, inherent to the minicircle network, may play an important role in creating a network topology simpler than expected. To test this hypothesis we provide a complete quantitative characterization of the effects induced by minicircle position, bending, electrostatic repulsion and orientation on the topological properties of the kDNA network. These factors are of immediate interest for understanding the formation of the kDNA network and have been qualitatively discussed in the literature [13, 14, 16, 17, 23, 24]. Our results show that, while all these factors are consistent with the observed experimental data, only minicircle orientation contributes significantly to the reduction of the predicted mean valence. Our findings are consistent with Delain and Riou’s proposed kDNA orientation model [23] based on observations of electron micrographs. In their model, minicircles are arranged almost perpendicular to the circular base of the kinetoplast disk (also known as the horizontal plane of the condensed kDNA disk). We propose that this arrangement of minicircles is the evolutionary solution in Kinetoplastids to the reduction of topological complexity induced by DNA condensation.

## Data, Assumptions and Modeling Methods

### 2.1 Physical characteristics of *C. fasciculata* kDNA networks

The mathematical models in this study are based on previously published data of the *C. fasciculata* kDNA network (see [13, 14] for reviews). In *C. fasciculata* about 5,000 2.5 kb relaxed minicircles are confined into the kinetoplast disk, a cylindrical structure.4 *μ*m in height and 1 *μ*m in diameter. Minicircles are organized into a planar topological network in which every pair of neighboring minicircles is singly linked by a Hopf link (see Fig 1A, [15]). In a non-replicating network, each minicircle is linked to an average of three other minicircles. The area of the horizontal plane of the condensed *in vivo* kDNA disk can be expressed in terms of the minicircle radius, and its value is about 47.94*r* ^2^, where *r* = .128*μ*m is the radius of a minicircle. Thus the density of minicircles in the kDNA network of *C. fasciculata* is about 104.3 minicircles per squared minicircle radius.

### 2.2 Biological and Mathematical Assumptions

The models described below are based on the following assumptions:

**Biological assumption 1**. The centers of the kDNA minicircles are distributed in the horizontal plane of the kDNA disk. This assumption is based on two experimental observations. First, the height of the kinetoplast disk is about half the length of the minicircles [14, 23, 25] and second, kDNA networks extracted from *C. fasciculata* are planar instead of three dimensional [15].

**Biological assumption 2**. Maxicircles do not determine the structure of the minicircle network. This assumption is based on experimental observations that the removal of maxicircles does not alter the structure of the network [16] and the spatial distribution of maxicircles is different for different organisms despite having the same minicircle topology [26].

**Mathematical assumption 1**. Minicircles have their centers at the vertices of the regular square lattice. This simplification is supported by the authors’ previous work [18, 27] that shows that the topological properties of the network do not change significantly when minicircles are placed on triangular or hexagonal lattices or even when they are randomly displaced from these lattices. We refer to such a lattice region with the minicircles placed in it as *minicircle grid* (See Fig 1B, 1C and 1D).

**Mathematical assumption 2**. Minicircles are represented by geometric flat circles (Fig 1B) or by flexible minicircles (Fig 1C). The use of rigid flat circles is justified by three observations: first kDNA minicircles are relaxed and not supercoiled [15], second they are linked by a single Hopf link (Fig 1A), the only possible link between two geometrical minicircles and third the linking probability of two flexible chains with a given radius of gyration can be seen as the linking probability of two rigid circles whose radius is the same value as the radius of gyration of the flexible chains with a noise component. We used freely jointed minicircles, instead of the more commonly used wormlike chain model [28], because the statistical properties of these protein-bound molecules may significantly deviate from those of the wormlike chain (Fig 1D) and because the bending of the wormlike chain [29] falls in between the freely jointed minicircles and the rigid minicircles even if localized sequence induced bending were to be introduced.

### 2.3 Quantitative description of minicircle networks

The *density of minicircles* is defined as the number of minicircles per squared radius of the minicircle (namely its length divided by 2*π*) and the density of minicircles is simply the reciprocal of this distance squared. A minicircle grid in which most minicircles are linked forming a topological network is called a *saturation network*, and the average density at which the saturation network forms is called *mean saturation density*. In our studies we measured saturation as a percentage to avoid artificially high saturation densities caused by minicircles along the grid boundary that have a smaller probability of linking than do minicircles within the grid. In this a network becomes saturated when 99% of the minicircles are linked together. The *mean valence of a minicircle* is the average valence value of a minicircle over the entire ensemble of possible minicircle grids. In all the calculations described below, unless stated otherwise, the mean saturation density was estimated by sampling 10,000 networks, and valence calculations for each density were performed by estimating the average number of minicircles linked to a non-boundary, randomly selected minicircle over 1,000 samples of 7 × 7 minicircle grids.

### 2.4 Modeling minicircle orientation

Methods for modeling orientation have been previously described [30]. The orientation of any minicircle is given by the orientation of the vector perpendicular to the plane containing the minicircle, which in turn is given by the the tilting and the azymuthal angles. In [30] we showed, through computer simulations, that restrictions on the tilting angle (1) can increase the density of minicircles while keeping the topology of the network simple and (2) preserve the linear relationship between density and mean valence. The azymuthal angle, on the other hand, showed highly nonlinear responses and was not considered biologically relevant. In this paper, we used the methods in [30] to estimate the effects of tilting angles that are consistent with experimental data.

### 2.5 Modeling the effects of DNA bending

We used freely-jointed minicircles to study the role of DNA bending (Fig 1C). This minicircle model reflects the effects of DNA sequence and/or histone-like proteins that condense DNA molecules hence reducing their overall radius of gyration and linking probability (Fig 1C). Methods have been reported elsewhere and will only be briefly reviewed here [31]. To generate grids of freely-jointed minicircles, we sampled minicircle conformations using the generalized hedgehog algorithm [32, 33], positioned them at the vertices of the lattice, and computed the linking number between pairs of neighboring minicircles following the Gaussian integral formulation [34]. In this study minicircles were represented by freely-jointed fragments. When modeling naked double stranded DNA in free solution one uses the value of 300*bp* per fragment (i.e. one Kuhn length). In this study we overemphasized the effects of chain bending by representing minicircles with twice the number of Kuhn lengths of the minicircle. Saturation densities were estimated on grids of size 100 × 100.

### 2.6 Modeling the effects of volume exclusion due to electrostatic repulsion of DNA chains

Minicircles with electrostatic volume exclusion were modeled by segmented polygons that traced the contour of the rigid minicircle (Fig 1D) or by freely jointed circular chains (Fig 1C); volume exclusion was modeled by an impenetrable cylinder around each segment of the polygon [35]. Two parameters defined these models: (1) the number of segments used to represent the DNA minicircle; and (2) the radius of the cylinder that defines the excluded volume (denoted by *τ*). These values were normalized by the length of the minicircle in order to compare them with experimental data.

We developed new algorithms to generate grids of minicircles with volume exclusion due to two important factors. First, the linking probability of two minicircles is not independent of whether other neighboring minicircles are topologically linked or not. This dependency is negligible in systems with no volume exclusion but it plays a key role otherwise. Second, previously defined algorithms mostly produce non-admissible conformations, due to the overlapping of minicircles. This algorithm, whose details are reported in a separate paper [36], had two stages. In the first stage we estimated the linking probability between two minicircles in small (10 × 10) grids and in the second stage we used the estimated linking probabilities and a ‘coin-tossing’ argument to generate large (1000 × 1000) grids.

Large samples of small minicircle grids were generated using a simulated annealing algorithm, since even for this small grids, methods used in our previous studies [18, 30] produced mostly unacceptable conformations. This algorithm starts with a minicircle grid, which may or may not be acceptable. The grid is assigned an energy penalty, given by the number of intersecting minicircles. If the initial energy is positive, a fixed number of minicircles are randomly selected and their orientations are randomized uniformly over all possible directions. The new configuration is accepted or rejected according to the standard Monte-Carlo criterion. This process terminates when a zero energy state is reached (meaning that an acceptable minicircle grid has been found), or when a pre-determined number of steps, between 300 and 1,000 (a value dependent on the density of minicircles), had occurred before reaching a lower energy state. The output of this algorithm is a large set of tables that contain the linking probability of two neighboring minicircles in the grid together with the topological state of other neighboring minicircles.

Next, we generated large grids that did not explicitly represent the minicircles but just their positions on the grid. To decide whether two neighboring minicircles in the large grid were topologically linked or not we first determined the topology of the nearby minicircles and looked for the table (produced in the first stage of the algorithm) that had the same combination of linked/unlinked neighboring minicircles. Once the table was identified the linking status of the two minicircles under consideration was decided using a ‘biased coin tossing’ algorithm with the linking probability given by the probability estimated in the first stage of the algorithm and given by the table.

## Numerical Results

### 3.1 Mathematical modeling revealed a discrepancy between predicted and observed minicircle density and mean minicircle valences

In our previous work we rigorously showed that, for any fixed minicircle grid size, a saturation network will form with a probability rapidly approaching 1 as the minicircle density increases [19]. Numerical results from these studies estimated that the mean saturation density is *D*_*Sat*_ ≈ 1.37 and its corresponding mean valence ≈ 4.84. The estimated *D*_*Sat*_ is therefore much smaller than the density of the kDNA network (≈ 104.3) and we can safely conclude, in agreement with experimental observations, that the kDNA network in *C. fasciculata* is saturated. There is however a significant discrepancy between the experimentally observed and the expected mean valences: at the density of ≈ 104.3, we predict a mean valence close to 400, while the experimentally determined valence in kDNA is only at 3 [17]. The observed differences are robust with respect to the relative position of minicircles in the grid [18, 27] thus validating our hypothesis and our use of the square lattice in this and other studies (Mathematical Assumption 1).

### 3.2 Neither DNA bending nor volume exclusion due to electrostatic interactions can account for the discrepancy between observed and predicted mean valences

As discussed in the methods section, the model that evaluates the effects of volume exclusion due to DNA electrostatics, has two parameters: (1) the number of segments representing the minicircle; and (2) the radius of the cylinder that defines the excluded volume. In [36] we concluded that (1) had negligible effects on the topology of the network and in this study we used a fixed value of 22 segments. The effects of (2) were estimated and revealed that saturation density increases linearly with the value of *τ* according to $D_{sat}^{\tau }=1.3511+8.2905\tau$, a value that is consistent with the calculated value for rigid minicircles for *τ* = 0 ($D_{sat}^{0}\approx 1.354$) [36]. Two values of the parameter *τ* were considered of biological relevance: *τ* ≈.008 and *τ* ≈.02. The value *τ* ≈.008 corresponds to 2nm and it is the geometric diameter of the DNA double helix. The value *τ* ≈.02 corresponds to 5nm and it is the effective diameter of the DNA double helix under physiological conditions (*i.e.* NaCl concentration of 0.15M) [35].

**Fig 2:**
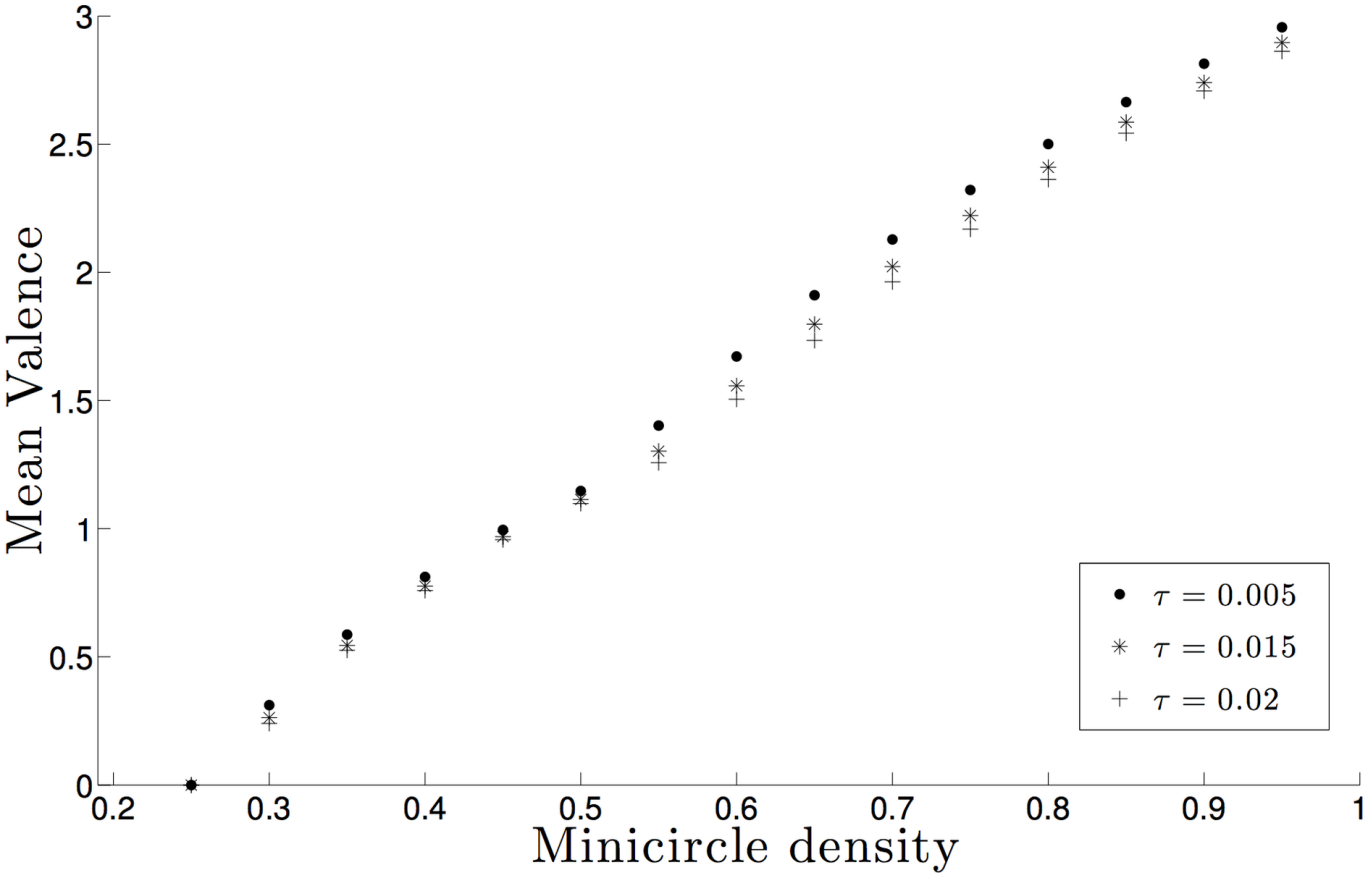
Estimation of the mean minicircle valence as a function of the minicircle density for biologically significant radii. The sample size for each data point is 10^5^ and the sizes of the error bars are less than the sizes of the plotted data points.

Fig 2 shows the relationship between minicircle density and mean valence for different excluded volumes. The figure clearly shows two important phenomena: first, there is a linear relationship between mean valence and minicircle density for each fixed *τ* value; second increasing minicircle volume exclusion reduces the value of the mean valence. The linear relationship between mean valence and minicircle density result is in good agreement with our previous studies [18, 19, 30] and therefore help validate the results presented here. The inverse relation between volume exclusion and mean valence supports the hypothesis that volume exclusion has a simplifying effect on the topology of the network [16]; interestingly both phenomena are conserved for moderate increases of the parameter *τ* [36] hence we conclude that our results do not change even if the salt conditions are somewhat different from those discussed above. Based on these results we argue that the topological simplification is very small and conclude that volume exclusion does not have leading role on determining the topology of the network.

We found significant differences when we took DNA bending into account. It is well known that factors such as stretches of specific DNA sequences [37] and DNA-protein interactions [38–40] can alter the overall shape of minicircles. To study DNA bending we used the freely-jointed model [41]. As described in the methods section, we represented each minicircle by a freely jointed polygonal chain with 16 edges. Using this model we estimated a mean saturation density of 3.04±.06 [31], a value not much larger than that observed for rigid minicircles. The mean valence at this density was ≈ 80 a significant decrease from 104.3 but not large enough to reach the experimentally observed values.

**Fig 3:**
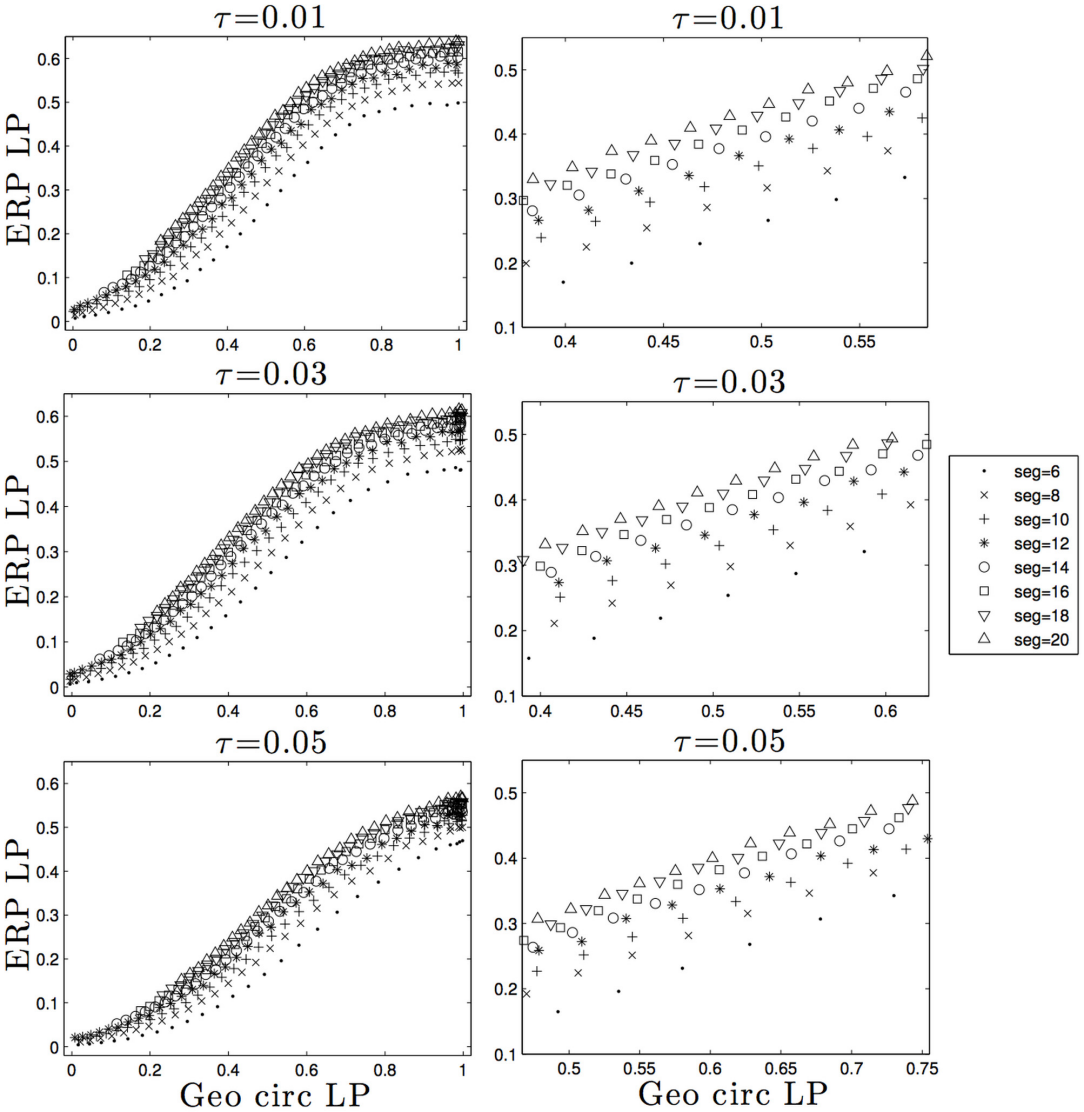
Relationship between the linking probability (LP) of rigid (Geo Circ) and freely jointed minicircles (ERP) with volume exclusion.

To test whether a combination of DNA bending and volume exclusion could have a significant effect on the mean valence of the network we added volume to the freely jointed minicircles. Instead of generating an entire network of minicircles, as in the previous studies, we compared the linking probabilities of two rigid minicircles and of two flexible minicircles with excluded volume. The radius of the rigid minicircles was equal to the radius of gyration of the flexile minicircles. We argue that if the linking probabilities between the two are similar then the induced network structure will not be too different. Results corresponding to these calculations are shown in Fig 3. The column on the left shows the comparison between the linking probabilities of the two models for different excluded volumes (*τ* = 0.01, 0.03 and 0.05) and different bending rigidities (*seg* = 6, ⋯, 20); the column on the right shows a magnification of the graph on the left for those linking probabilities for which the network forms. As expected the geometric minicircle (x-axis) reaches a linking probability equal to one faster than flexible minicircles; however there is a strong linear relationship for most of the compared values (left column) which is robust with respect to the bending rigidity of the minicircle. This linear relationship is due to the fact that the linking probability of two freely jointed chains is closely related to the linking probability of two minicircles whose radii are equal to the radii of gyration of the corresponding freely jointed chains.

Our results are consistent but not necessarily directly comparable to some previously published results. In particular, our results for the linking probability between two minicircles presented here and in [31] are similar to those presented in [42] except that our simulated molecules are much smaller and thinner than those in [42]. For instance, the value for the linking probability between two random chains with overlapping centers of masses, the decaying trend of the linking probability as a function of the distance between the center of masses between two chains and the change in linking probability for the thickness studied here are consistent among these three studies (see Fig 5a and 5b in [42] and Fig 6 in [31]).

Based on these results we therefore suggest that adding volume exclusion to freely jointed minicircles simplifies the topology of the network but, as argued above, the effects are not strong enough to significantly decrease the value of the mean valence from 80 to near 3.

### 3.3 The relative orientation of minicircles significantly decreases mean valence and explains most of the discrepancy between observations and predictions

**Fig 4:**
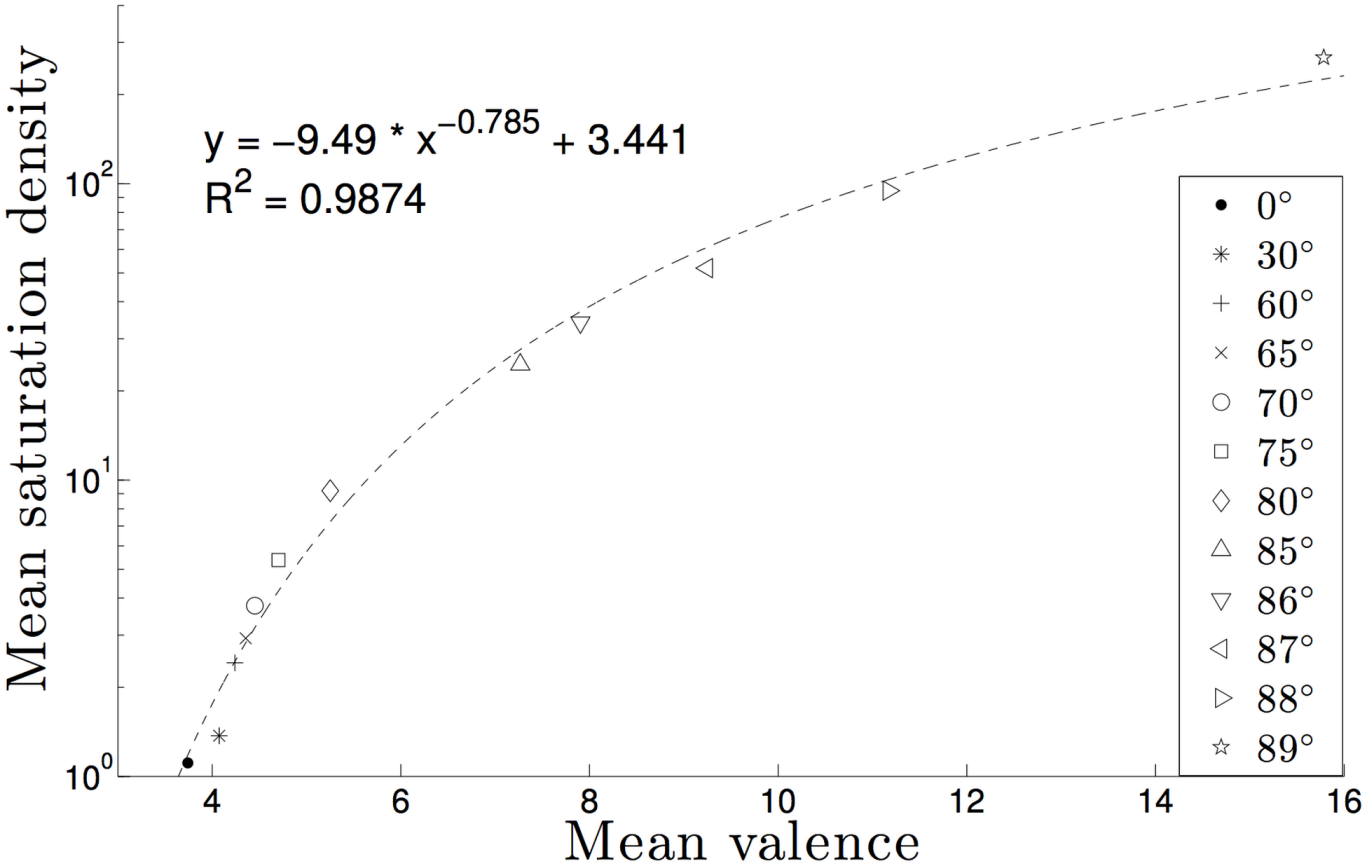
Estimated average saturation density for various restriction angles. Each data point in the figures is based on samples of sample size 1000 and minicircle grids of dimension 1000 × 1000. The 95% standard error bars are less than.0005 in all cases.

Experimental studies show that the height of the kinetoplast disk is roughly half of the length of the minicircle length and that minicircles are aligned almost perpendicular to the horizontal plane of the kDNA disk [23, 24]. In a previous study we characterized the effect of minicircle orientation on the topological properties of minicircle grids [30]. We therefore extended our previous study by sampling tilting values near 90 degrees. Clearly the tilting angle cannot be exactly 90 degrees because minicircles cannot be linked in this situation even if the azimuthal angle has no restrictions. Numerical estimations of the mean saturation densities, as well as the corresponding mean valences at these densities, are plotted in Fig 4. The angle that best represents the observed experimental data was 88 degrees. At this angle, the mean saturation density is 94.74 with a mean valence of 11.17. These values are within the same order of magnitude of the minicircle density and mean valence observed experimentally in a kDNA network and less than two fold of the replicated network.

## Conclusion

Mitochondrial DNA from most Kinetoplastids is composed of numerous minicircles and maxicircles with a diversity of kDNA organization [4, 7, 43, 44] Interestingly, no free-living organism in the group contains a large network of topologically linked molecules [9]. The establishment of the kDNA network has been a topic of interest and various hypotheses have been proposed with confinement being the prevailing one of biophysical nature (see [9, 13, 14] for reviews). Although this hypothesis is supported by indirect experimental results that show that the topological complexity of single and multiple circular chains increases in the presence of condensing agents [45–51] it remains to be determined how to directly test this hypothesis *in vivo* since disruption of the kDNA structure results in loss of fitness (reviewed in [13]).

In our previous work [19] we used mathematical modeling of the *C. fasciculata* kDNA network to test for confinement. Our results, in agreement with experimental data, supported the confinement hypothesis and showed that a network is the most likely conformation when minicircles are in a confined volume and that in confined conditions the mean minicircle valence increases linearly with minicircle density. This second observation is particularly relevant since it is in close agreement with the observed changes in the topology of the kDNA network during the cell cycle [16]. Our mathematical modeling approach also estimated, for the first time, the variance of the mean valence and predicted a critical density threshold in the evolution of kDNA network structure. More specifically our results suggest that during the evolution of these organisms, minicircles became highly concentrated to the point that a network was highly likely provided that dsDNA passage was permitted due to the action of topoisomerases. This phenomenon has indeed been observed in *in vitro* assays [47, 50, 51]. Once the network was first formed, its topology became a key biological component since very complex topology, a natural byproduct of higher DNA concentrations, would obstruct essential biological functions of the mitochondrion. Interestingly the formation of the network and its topology became essential for some organisms (see [13] for a review).

One would intuitively predict that the topology of the network is very complex since 5,000 minicircles of 0.5 *μ*m in diameter are condensed into a horizontal disk that is just 1 *μ*m in diameter. This intuitive argument is at odds with experimental results that show that the mean minicircle valence in kDNA is only three. How can the topology of the network remain so simple given the high density of minicircles? In this study we tested the most plausible biophysical factors that may contribute to maintain the simple topology. We first tested those factors that have been previously proposed in the most recent literature [17]: DNA bending induced by DNA sequence or DNA binding proteins and volume exclusion induced by electrostatics. Our results show that neither factor can account for the observed mean valence *in vivo*. On the other hand we found that the proposed positioning of minicircles by Delain and Riou [23], in which DNA filaments appear to be preferentially oriented to the height of the disk, can bring the value of the mean valence closer the value observed experimentally. We therefore propose that the topological simplicity of minicircle networks is mostly controlled by the orientation of minicircles and modulated to a lesser extent by DNA bending and volume exclusion among other factors.

One may wonder what are the mechanisms keeping minicircles in such position. Experimental evidence suggest that the Tripartite Attachment Complex [52], a set of filaments that connect the flagellar basal body to the kDNA, may have an important role on this positioning and in preserving a simplified topology. In fact, mutations to proteins of this complex [53, 54] show enlarged and potentially more complicated networks. Histone-like kinetoplast associated proteins (KAP) may also be involved in the positioning of minicircles since they are implicated in condensing the kDNA network [55–58] and in the remodeling of the kDNA structure during different life cycle phases [59]. Recent experimental works show that these proteins decrease the excluded volume effects of the minicircles and that interaction of KAP3 and KAP4 with the initiator protein, universal minicircle sequence-binding protein, results in decondensation of the kDNA network and increasing accessibility to topoisomerase II. Furthermore a recent report also suggest that down regulation of KAP6 is associated with the disorganization and loss of the kDNA network suggesting a potential role of this protein in replication and maintenance of the network [60]. Finally it is also possible that the position of some minicircles is not directly determined by a protein complex but instead by some neighboring minicircles, since it is well known that at high concentrations DNA molecules align parallel to each other (e.g., [61, 62]).

Our proposed arrangement of minicircles, with a tilting angle of about 88 degrees with respect to the horizontal plane of the condensed kDNA disk, produces a network that is consistent with the available experimental data. We however believe that there is still room for improvement. For instance our results above show a mean valence that is very close to that observed in nature (in particular it is less than 2 fold the value of the replicated network) but still larger than the value observed in the pre-replicated network. At this point many models, driven by the interplay between DNA bending and volume exclusion, could explain the difference between the observed and predicted mean valence. For instance, one could argue that directed, rather than random bending, could reduce this difference. This hypothesis is consistent with the fact that most minicircles have a region of bent DNA, its biological or structural functions however are not currently known [63]. A second argument would concern the effects of excluded volume due to the ionic conditions of the environment or to DNA bound proteins. In this study we are reporting results for excluded volume values of naked DNA in a standard range of *in vitro* conditions. One would expect the excluded volume to have a larger role at higher minicircle densities or when extreme tilting angles are taken into consideration. These effects however could be counterbalanced by the binding of KAP proteins to the DNA molecules. Implementation of these models would add a fairly large number of parameters and at the same time they would be difficult to validate since there is very little available experimental data.

We would like to emphasize that our model, in its current form, is not intended for representing the biological mechanism of network formation since it is statistical in nature and not dynamic. Our proposed arrangement of minicircles however can be used for developing dynamic models of kDNA network replication and maintenance.

## References

[1] World Health Organization. Research Priorities for Chagas Disease, Human African Trypanosomiasis and Leishmaniasis. World Health Organ Tech Rep Ser. 2012; 975: v–xii. 23484340

[2] Stuart K, Brun R, Croft S, Fairlamb A, Gurtler RE, McKerrow J et al Kinetoplastids: related protozoan pathogens, different disease. J Clin Invest. 2008; 118: 1301–1310. 10.1172/JCI33945 18382742 PMC2276762

[3] Benne R, Van den Burg J, Brakenhoff JP, Sloof P, Van Boom JH, Tromp MC. Major transcript of the frameshifted coxii gene from trypanosome mitochondria contains four nucleotides that are not encoded in the DNA. Cell. 1986; 46: 819–826. 10.1016/0092-8674(86)90063-2 3019552

[4] Lukes J, Guilbride DL, Votypka J, Zíkova A, Benne R, Englund PT. Kinetoplast DNA network: Evolution of an Improbable Structure. Euk Cell. 2002; 1: 495–502. 10.1128/EC.1.4.495-502.2002 PMC117999 12455998

[5] Simpson L, Sbicego S, Aphasizhev R. Uridine insertion/deletion RNA editing in trypanosome mitochondria: a complex business. RNA. 2003; 9: 265–276. 10.1261/rna.2178403 12591999 PMC1370392

[6] Stuart KD, Schnaufer A, Ernst NL, Panigrahi AK. Complex management: RNA editing in trypanosomes. Trends Biochem Sci. 2005; 30: 97–105. 10.1016/j.tibs.2004.12.006 15691655

[7] Maslov DA, Simpson L. RNA editing and mitochondrial genomic organization in the Cryptobiid Kinetoplastid Protozoan *Trypanoplasma borreli* . Mol Cell Biol. 1994; 14: 8174–8182. 7969154 10.1128/mcb.14.12.8174-8182.1994 PMC359356

[8] Yasuhira S and Simpson L. Guide RNAs and guide RNA genes in the cryptobiid kinetoplastid protozoan *Trypanoplasma borreli* . RNA. 1996; 2: 1153–1160 8903345 PMC1369444

[9] Lukes J, Skalicky T, Tyc J, Votypka J, Yurchenko V. Evolution of parasitism in kinetoplastid flagellates. Mol Biochem Parasitol. 2014; 195: 115–122. 10.1016/j.molbiopara.2014.05.007 24893339

[10] Roy Chowdhury A, Bakshi R, Wang J, Yildirir G, Liu B, Pappas-Brown V et al The killing of African trypanosomes by ethidium bromide. PLoS Pathog. 2010; 6(12): e1001226 10.1371/journal.ppat.1001226 21187912 PMC3002999

[11] Balaña-Fouce R, Alvarez-Velilla R, Fernández-Prada C, García-Estrada C, Reguera RM. Trypanosomatids topoisomerase re-visited. New structural findings and role in drug discovery. Int J Parasitol Drugs Drug Resist. 2014; 4(3): 326–37. 10.1016/j.ijpddr.2014.07.006 25516844 PMC4266802

[12] Zuma AA, Cavalcanti DP, Zogovich M, Machado AC, Mendes IC, Thiry M et al Unveiling the effects of berenil, a DNA-binding drug, on *Trypanosoma cruzi*: implications for kDNA ultrastructure and replication. Parasitol Res. 2015; 114(2): 419–30. 10.1007/s00436-014-4199-8 25349143

[13] Jensen RE, Englund PT. Network news: the replication of kinetoplast DNA. Annu Rev Microbio. 2012; 66: 473–491. 10.1146/annurev-micro-092611-150057 22994497

[14] Shapiro TA, Englund PT. The structure and replication of kinetoplast DNA. Annu Rev Microbiol. 1995; 49: 117–143. 10.1146/annurev.mi.49.100195.001001 8561456

[15] Rauch CA, Perez-Morga D, Cozzarelli NR, Englund PT. The absence of supercoiling in kinetoplast DNA minicircles. EMBO J. 1993; 12: 403–411. 8382606 10.1002/j.1460-2075.1993.tb05672.x PMC413223

[16] Chen J, Englund PT, Cozzarelli NR. Changes in network topology during the replication of kinetoplast DNA. EMBO J. 1995; 14: 6339–6347. 8557054 10.1002/j.1460-2075.1995.tb00325.x PMC394759

[17] Chen J, Rausch CA, White JH, Englund PT, Cazzarelli NR. The topology of the kinetoplast DNA network. Cell. 1995; 80: 61–69. 10.1016/0092-8674(95)90451-4 7813018

[18] Diao Y, Hinson K, Arsuaga J. The growth of minicircle networks on regular lattices. J Phys A Math Theory. 2012; 45(3): 035004 10.1088/1751-8113/45/3/035004

[19] Diao Y, Hinson K, Kaplan R, Vazquez M, Arsuaga J. The effects of minicircle density on the topological structure of mitochondrial DNA from trypanosomes. J Math Biol. 2012; 64: 1087–1108. 10.1007/s00285-011-0438-0 21671031

[20] Rybenkov VV, Vologoskii AV, Cozzarelli NR. The effect of ionic conditions on the conformations of supercoiled DNA. I. Sedimentation analysis. J Mol Biol. 1997; 267: 299–311. 10.1006/jmbi.1996.0877 9096227

[21] Wang Z, Englund PT. RNA interference of a trypanosome topoisomerase II causes progressive loss of mitochondrial DNA. EMBO J. 2001; 20: 4674–4683. 10.1093/emboj/20.17.4674 11532932 PMC125608

[22] Lindsay ME, Gluenz E, Gull K, Englund PT. A new function of *Trypanosoma brucei* mitochondrial topoisomerase II is to maintain kinetoplast DNA network topology. Mol Microbio. 2008; 70: 1465–1476. 10.1111/j.1365-2958.2008.06493.x PMC2993328 19019151

[23] Delain E, Riou G. DNA ultrastructure of the kinetoplast of *Trypanosoma cruzi* cultivated *in vitro* . C R Acad Sci Hebd Seances Acad Sci. 1969; 268: 1225–1227. 4975253

[24] Renger HC, Wolstenholme DR. The form and structure of kinetoplast DNA of *Crithidia* . J Cell Biol. 1972; 54(2): 346–364. 10.1083/jcb.54.2.346 5040863 PMC2108870

[25] Simpson L. The kinetoplast of the hemoflagellates. Int Rev Cytol. 1972; 32: 139–207.

[26] Ferguson ML, Torri AF, Ward DC, Englund PT. *In situ* hybridization to the *Crithidia fasciculata* kinetoplast reveals two antipodal sites involved in kinetoplast DNA replication. Cell. 1992; 70: 621–629. 10.1016/0092-8674(92)90431-B 1324122

[27] Rodrigues V, Diao Y, Arsuaga J. Percolation phenomena in disordered topological networks. J Phys A Math Theory. 2013; 45(4): 012070.

[28] Vologodskii A. Simulations of equilibrium and dynamic properties of large DNA molecules. In Computational Studies of RNA and DNA, Springer. 2006; 579–604. 10.1007/978-1-4020-4851-3_23

[29] Vologodskii AV, Rybenkov V. Simulation of DNA catenanes. Chem Phys. 2009; 11: 10543–10552. 10.1039/b910812b PMC2845312 20145800

[30] Arsuga J, Diao Y, Hinson K. The effect of angle restriction on the topological characteristics of minicircle networks. J Statist Phys. 2012; 146: 434–445. 10.1007/s10955-011-0386-5

[31] Arsuaga J, Diao Y, Klingbeil M, Rodriguez V. Properties of topological networks of flexible polygonal chains. Mol Based Math Biol. 2014; 2: 98–106. 10.2478/mlbmb-2014-0007

[32] Scharein RG. *KnotPlot*, a program for drawing, visualizing, manipulating, and energy minimizing knots. Available: http://www.knotplot.com.

[33] Varela R, Hinson K, Arsuaga J, Diao Y. A fast ergodic algorithm for generating ensembles of equilateral random polygons. J Phys A Math Theory. 2009; 42(9): 095204 10.1088/1751-8113/42/9/095204

[34] Klenin K, Langowski J. Computation of writhe in modeling of supercoiled DNA. Biopolymers. 2000; 54: 307–317. 10.1002/1097-0282(20001015)54:5<307::AID-BIP20>3.0.CO;2-Y 10935971

[35] Rybenkov VV, Cozzarelli NR, Vologodskii AV. Probability of DNA knotting and the effective diameter of the DNA double helix. Proc Natl Acad Sci USA. 1993; 90: 5307–5311. 10.1073/pnas.90.11.5307 8506378 PMC46705

[36] Diao Y, Hinson K, Arsuaga J. The effect of volume exclusion on the formation of DNA minicircle networks. 2015; Preprint.

[37] Marini JC, Levine SD, Crothers DM, Englund PT. A bent helix in kinetoplast DNA. Cold Spring Harb Symp Quant Biol. 1983; 47: 279–283. 10.1101/SQB.1983.047.01.033 6574847

[38] Abu-Elneel K, Kapeller I, Shlomai J. Universal minicircle sequence-binding protein, a sequence-specific DNA-binding protein that recognizes the two replication origins of the kinetoplast DNA minicircle. J. Biol Chem. 1999; 274: 13419–13426. 10.1074/jbc.274.19.13419 10224106

[39] Avrahami D, Tzfati Y, Shlomai J. A single-stranded DNA binding protein binds the origin of replication of the duplex kinetoplast DNA. Proc Natl Acad Sci USA. 1995; 92: 10511–10515. 10.1073/pnas.92.23.10511 7479830 PMC40641

[40] Hines JC, Ray DS. The *Crithidia fasciculata* KAP1 gene encodes a highly basic protein associated with kinetoplast DNA. Mol Biochem Parasitol. 1998; 94: 41–52. 10.1016/S0166-6851(98)00048-6 9719509

[41] Cantor CR, Schimmel PR. Biophysical Chemistry, Pat II: Techniques for the study of Biological structure and Function. 1980; Macmillan.

[42] Hirayama H, Tsurusaki K, Deguchi T. Linking probabilities of off-lattice self-avoiding polygons and the effects of excluded volume. J Phys A Math Theory. 2009; 42: 105001 10.1088/1751-8113/42/10/105001

[43] Flegontov P, Votýpka J, Skalický T, Logacheva MD, Penin AA, Tanifuji G et al *Paratrypanosoma* is a novel early-branching trypanosomatid. Curr Biol. 2013; 23(18): 1787–1793. 10.1016/j.cub.2013.07.045 24012313

[44] Lukescaron J, Jirkû M, Avliyakulov N, Benada O. Pankinetoplast DNA structure in a primitive bodonid flagellate, *Cryptobia helicis* . EMBO J. 1998; 17(3): 838–846. 10.1093/emboj/17.3.838 9451008 PMC1170432

[45] Arsuaga J, Vasquez M, Trigueros S, Sumners D, Roca J. Knotting probability of DNA molecules confined in restricted volumes: DNA knotting I phage capsids. Proc Natl Acad Sci USA. 2002; 99: 5373–5377. 10.1073/pnas.032095099 11959991 PMC122776

[46] Cejka P, Plank JL, Dombrowski CC, Kowalczykowski SC. Decatenation of DNA by the S. Cerevisiae Sgs1-Top3-Rmi1 and RPA complex: a mechanism for disentangling chromosomes. Mol Cell. 2012; 47: 886–896. 10.1016/j.molcel.2012.06.032 22885009 PMC3462259

[47] Harmon FG, Brockman JP, Kowalczykowski SC. RecQ helicase stimulated both DNA catenation and changes in DNA topology by topoisomerase III. J Biol Chem. 2003; 278: 42668–42678. 10.1074/jbc.M302994200 12909639

[48] Hsieh T. Knotting of the circular duplex DNA by type II DNA topoisomerase from *Drosophila melanogaster* . J Biol Chem. 1983; 258: 8413–9420. 6305983

[49] Kimura K, Rybenkov VV, Crisona NJ, Hirano T, Cozzarelli NR. 13S condensing actively reconfigures DNA by introducing global positive writhe: implications for chromosome condensation. Cell. 1999; 98: 239–248. 10.1016/S0092-8674(00)81018-1 10428035

[50] Kreuzer KN, Cozzarelli NR. Formation and resolution of DNA catenanes by DNA gyrase. Cell. 1980; 20: 245–254. 10.1016/0092-8674(80)90252-4 6248235

[51] Waldeck W, Theobald M, Zentgraf H. Catenation of DNA by eukcaryotic topoisomerase II associated with simian virus 40 minichromosomes. EMBO J. 1983; 2: 1255–1261. 10872317 10.1002/j.1460-2075.1983.tb01578.x PMC555269

[52] Ogbadoyi EO, Robinson DR, Gull K. A high-order trans-membrane structural linkage is responsible for mitochondrial genome positioning and segregation by flagellar basal bodies in trypanosomes. Mol Biol Cell. 2003; 14: 1769–1779. 10.1091/mbc.E02-08-0525 12802053 PMC165075

[53] Schnarwiler F, Niemann M, Doiron N, Harsman A, Käser S, Mani J et al Trypanosomal TAC40 constitutes a novel subclass of mitochondrial beta-barrel proteins specialized in mitochondrial genome inheritance. Proc Natl Acad Sci USA. 2014; 111: 7624–7629. 10.1073/pnas.1404854111 24821793 PMC4040615

[54] Zhao Z, Lindsay ME, Chowdhury AR, Robinson DR, Englund PT. p166, a link between the trypanosome mitochondrial DNA and flagellum, mediates genome segregation. EMBO J. 2008; 27: 143–154. 10.1038/sj.emboj.7601956 18059470 PMC2206137

[55] Avliyakulov NK, Lukes J, Ray DS. Mitochondrial histone-like DNA-binding proteins are essential for normal cell growth and mitochondrial function in *Crithidia fasciculata* . Eukaryot Cell. 2004; 3: 518–526. 10.1128/EC.3.2.518-526.2004 15075280 PMC387644

[56] Lukes J, Hines JC, Evans CJ, Avliyakulov NK, Prabhu VP, Chen J et al Disruption of the *Crithidia fasciculata* KAP1 gene results in structural rearrangement of the kinetoplast disc. Mol Biochem Parasitol. 2001; 117: 179–186. 10.1016/S0166-6851(01)00348-6 11606228

[57] Onn I, Kapeller I, Abu-Elneel K, Shlomai J. Binding of the universal minicircle sequence binding protein at the kinetoplast DNA replication origin. J Biol Chem. 2006; 281: 37468–37476. 10.1074/jbc.M606374200 17046830

[58] Kapeller I, Milman N, Yaffe N, Shlomai J. Interactions of a replication initiator with histone H1-like proteins remodel the condensed mitochondrial genome. J Biol Chem. 2011; 286: 40566–40574. 10.1074/jbc.M111.270322 21984849 PMC3220483

[59] Cavalcanti DP, Shimada MK, Probst CM, Souto-Padrón TC, de Souza W, Goldenberg S et al Expression and subcellular localization of kinetoplast-associated proteins in the different developmental stages of Trypanosoma cruzi. BMC Microbiol. 2009; 9: 120 10.1186/1471-2180-9-120 19497120 PMC2700280

[60] Wang J, Pappas-Brown V, Englund PT, Jensen RE. TbKAP6, a mitochondrial HMG box-containing protein in Trypanosoma brucei, is the first trypanosomatid kinetoplast-associated protein essential for kinetoplast DNA replication and maintenance. Eukaryot Cell. 2014; 13: 919–932. 10.1128/EC.00260-13 24879122 PMC4135736

[61] Leforestier A, Livolant F. Supramolecular ordering of DNA in the cholesteric liquid crystalling phase: an ultrastructural study. Biophys J. 1993; 65: 56–72. 10.1016/S0006-3495(93)81063-4 8369461 PMC1225700

[62] Pelta J Jr., Durand D, Doucet J, Livolant F. DNA mesophases induced by spermidine: structural properties and biological implications. Biophys J. 1996; 71: 48–63. 10.1016/S0006-3495(96)79232-9 8804588 PMC1233456

[63] Englund PT. A passion for parasites. J Biol Chem. 2014; 289(49): 33712–33729. 10.1074/jbc.X114.620666 25336639 PMC4256308

